# Crystal Structures of Two Titanium Phosphate-Based Proton Conductors: Ab Initio Structure Solution and Materials Properties

**DOI:** 10.1021/acs.inorgchem.1c02613

**Published:** 2021-11-22

**Authors:** Hilke Petersen, Niklas Stegmann, Michael Fischer, Bodo Zibrowius, Ivan Radev, Wladimir Philippi, Wolfgang Schmidt, Claudia Weidenthaler

**Affiliations:** † Heterogeneous Catalysis, Max-Planck-Institut für Kohlenforschung, Kaiser-Wilhelm-Platz 1, 45470 Mülheim an der Ruhr, Germany; ‡ MAPEX Center for Materials and Processes, University of Bremen, 28334 Bremen, Germany; § Crystallography/Geosciences, University of Bremen, Klagenfurter Straße, 28359 Bremen, Germany; ∥ The Hydrogen and Fuel Cell Center ZBT GmbH, Carl-Benz-Straße 201, 47057 Duisburg, Germany

## Abstract

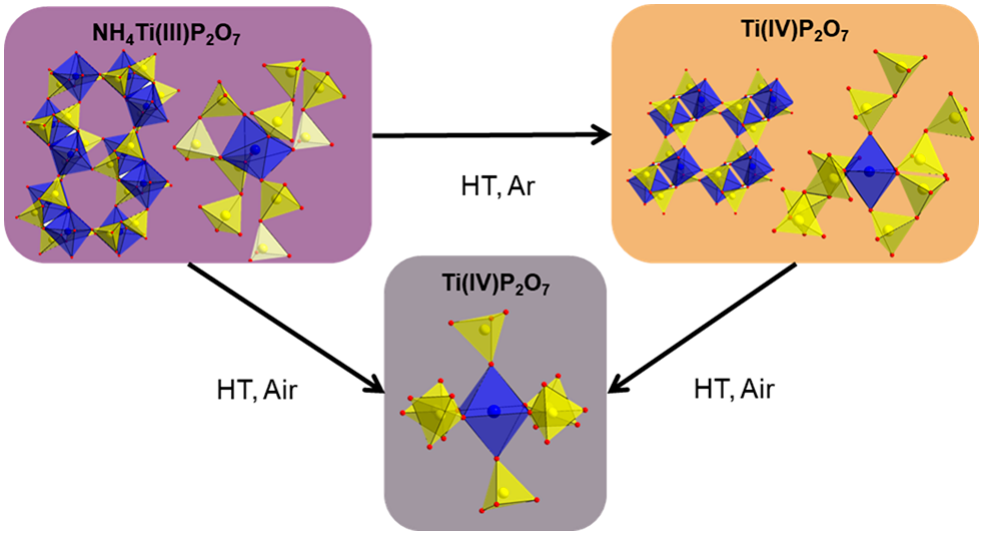

Transition-metal phosphates show a wide range of chemical compositions, variations of the valence states, and crystal structures. They are commercially used as solid-state catalysts, cathode materials in rechargeable batteries, or potential candidates for proton-exchange membranes in fuel cells. Here, we report on the successful ab initio structure determination of two novel titanium pyrophosphates, Ti(III)p and Ti(IV)p, from powder X-ray diffraction (PXRD) data. The low-symmetry space groups *P*2_1_/*c* for Ti(III)p and *P*1̅ for Ti(IV)p required the combination of spectroscopic and diffraction techniques for structure determination. In Ti(III)p, trivalent titanium ions occupy the center of TiO_6_ polyhedra, coordinated by five pyrophosphate groups, one of them as a bidentate ligand. This secondary coordination causes the formation of one-dimensional six-membered ring channels with a diameter *d*_max_ of 3.93(2) Å, which is stabilized by NH_4_^+^ ions. Annealing Ti(III)p in inert atmospheres results in the formation of a new compound, denoted as Ti(IV)p. The structure of this compound shows a similar three-dimensional framework consisting of [PO_4_]^3–^ tetrahedra and Ti^IV+^O_6_ octahedra and an empty one-dimensional channel with a diameter *d*_max_ of 5.07(1) Å. The *in situ* PXRD of the transformation of Ti(III)p to Ti(IV)p reveals a two-step mechanism, i.e., the decomposition of NH_4_^+^ ions in a first step and subsequent structure relaxation. The specific proton conductivity and activation energy of the proton migration of Ti(III)p, governed by the Grotthus mechanism, belong to the highest and lowest, respectively, ever reported for this class of materials, which reveals its potential application in electrochemical devices like fuel cells and water electrolyzers in the intermediate temperature range.

## Abstract 2

The crystal structures of two novel transition-metal phosphates were solved via the combination of spectroscopic and diffraction methods. The reaction mechanism of the transformation of the structures was studied *in situ*. Additionally, the proton conductivity of the compounds was analyzed.

## Introduction

**Figure 1:**
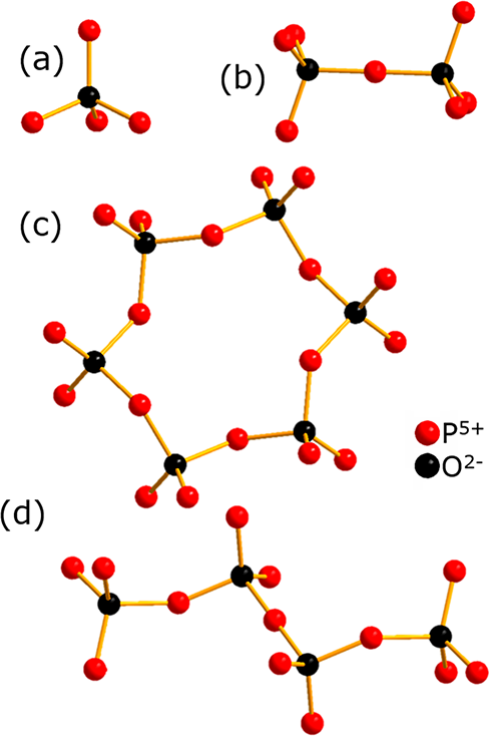
Examples of the different classes of condensed phosphates: (a) orthophosphate, (b) pyrophosphate, (c) ring, and (d) chain metaphosphates. The classification is according to Popović et al.^25^

Transition-metal phosphates (TMPs) are a class of functional materials that are not only studied for fundamental understanding but also applied in industrial applications.^1−8^ The members of the TMP family show a wide range of chemical compositions and a wide variety of crystal structures with variable metal coordination and different phosphate structure units. The phosphate units are differentiated in orthophosphates ([PO_4_]^3–^) and different condensed phosphates, pyrophosphates ([P_2_O_7_]^4–^) and metaphosphates, consisting either of [PO_3_]^−^_*n*_ chains or [P_*n*_O_3*n*_]^*n*−^ rings (Figure 1). The structural properties of TMPs determine their potential as efficient and environmentally sustainable cathode materials in rechargeable batteries.^3,5−7,9−11^ The orthophosphate LiFePO_4_ is a well-known example for a successful energy storage material showing high capacity and charge–discharge reversibility, together with the economic requirements of low cost and environmental friendliness.^3,7,10,12−14^ Among TMPs, vanadyl pyrophosphate [VO(P_2_O_7_)] is especially interesting because it is the only commercially used solid-state catalyst for the selective oxidation of butane to maleic anhydride.^1,4,8,15^*Operando* and *in situ* Raman studies of the activation of the hemihydrate VOHPO_4_·0.5H_2_O to the active VO(P_2_O_7_) revealed a complex activation process including different reorganization processes on the crystallite surface and in the crystalline bulk material.^1,4^

Titanium phosphates belong to the intensively studied TMP compounds. They show promising behavior as photocatalysts, solid acids, N_2_ absorbents in the Haber–Bosch process, and proton conductors, in particular in the mediate temperature range.^5,6,16,17^ The wide application range of titanium phosphates is caused by their structural variety. The structure of monoclinic metaphosphate Ti(PO_3_)_3_ is formed by isolated [TiO_6_]^3–^ octahedra connected with [PO_3_]^−^_*n*_ tetrahedral (*n* = 3, 6) *zigzag* chains propagating along the crystallographic *a* direction.^18^ The orthorhombic titanium phosphate TiPO_4_ crystallizes in the space group *Cmcm* and is formed by chains of edge-sharing TiO_6_ octahedra connected via corner-sharing [PO_4_]^3–^ groups propagating along the crystallographic *c* axis.^19^ The orthophosphate TiPO_4_ shows an interesting structural behavior if pressurized in a diamond anvil cell. Above 46 GPa, the phosphorus atom is coordinated by five oxygen atoms, forming a chain of trigonal bipyramids [PO_5_]^5–^ along the [101] direction.^20^ Two open-framework structures, Ti_2_O(PO_4_)_2_·2H_2_O, and its dehydrated form, Ti_2_O(PO_4_)_2_, are also formed by [PO_4_]^3–^ and TiO_6_ polyhedra.^16,21^ Both structures contain two one-dimensional chains, [P_*n*_O_3*n*_]^*n*−^, along the crystallographic *a* axis with *n* = 6 and 8.^16,21,22^ In Ti_2_O(PO_4_)_2_·2H_2_O, one of the two independent titanium sites is coordinated by two water molecules, forming a highly distorted TiO_6_ octahedron. In the dehydration process, the titanium coordination changes to a TiO_4_ tetrahedron, creating new acidic sites on the surface of the particle.^16^

Titanium pyrophosphate along with other metal pyrophosphates, MP_2_O_7_ (M = Sn, Ti, Si, Ge, Zr, Ce), is a potential candidate for proton-exchange membranes in next-generation fuel cells, working in the intermediate temperature range.^5,17^ Their proton conductivity at temperatures between 273 and 673 K under water-free conditions makes these compounds particularly suitable for this application.^5^ Most MP_2_O_7_ compounds (M = Si, Sn, Pb, Ti, Zr, Hf, U, Ce) can be described via a cubic parent structure with the formula unit *Z* = 4 and lattice parameter *a* ≈ 8 Å but show a superstructure similar to that of a cubic 3 × 3 × 3 supercell with *Z* = 108.^23^ The first description of the TiP_2_O_7_ structure by Levi and Peyronel obtained in a powder X-ray diffraction (PXRD) study resulted also in a small cubic unit cell in *Pa*3̅ with *a* = 7.80(1) Å. Recent PXRD and single-crystal structure analyses showed that TiP_2_O_7_ consists of a cubic superstructure in *Pa*3̅ with lattice parameter *a* = 23.6383(2) Å.^23,24^ In both proposed structure models, titanium is coordinated in a quite regular TiO_6_ octahedron with typical Ti–O bond lengths [*r*(Ti–O) = 1.88(2)–1.98(2) Å] and O–Ti–O angles close to 90°.^23,24^ In the structure model reported by Levi and Peyronel, all pyrophosphate groups occupy positions on the 3-fold rotation axis. In the superstructure, four of the six independent pyrophosphate groups show P–O–P angles between 139(1) and 145(1)° and between 141.5(1) and 144.5(1)°.^23,24^ The P–O_B_ (with O_B_ as the bridging oxygen atom in the pyrophosphate group) bond distances of the bent pyrophosphates show a variety of possible bond lengths [*r*(P–O_B_) = 1.57(1)–1.60(1) Å].^23,24^

In a recent publication, we introduced a novel synthesis route for TMPs using metal oxides and NH_4_(H_2_PO_2_) as a phosphorus source instead of phosphate-based precursors.^26^ The specific feature of this synthesis route is the reducing property of NH_4_(H_2_PO_2_), which stabilizes low-valent transition-metal compounds. With this synthesis route, two novel titanium phosphates with unknown structures could be obtained, hereafter referred to as Ti(III)p and Ti(IV)p. Ti(III)p is formed by annealing the reaction mixture to 573 K. Interestingly, this new phase contains trivalent titanium cations.^26^ The combination of the analytical results obtained by energy-dispersive X-ray spectroscopy and X-ray photoelectron spectroscopy (XPS) and analysis of the gaseous decomposition products via thermogravimetric analysis/differential scanning calorimetry coupled with mass spectroscopy (TGA/DSC–MS) result in the chemical composition NH_4_Ti^III+^P_2_O_7_. A preliminary discussion of the Raman spectra implies the existence of pyrophosphates [P_2_O_7_]^4–^ in the structure.^26^ At elevated temperatures, Ti(III)p reacts to the well-known cubic TiP_2_O_7_ structure in air.^26^ On the contrary, annealing Ti(III)p in inert atmospheres causes the formation of another yet unknown titanium phosphate structure (Ti(IV)p).^26^ In the TGA/DSC–MS experiment, two endothermic signals at ∼723 and ∼762 K are observed during this reaction. Both signals correlate with the decomposition of NH_4_^+^ and the release of hydrogen (H_2_) and ammonia (NH_3_), as evidenced by the MS data.^26^ The XPS data of Ti(IV)p show, in contrast to Ti(III)p, solely Ti in the oxidation state IV+.^26^ The H_2_ release and the oxidation of Ti^3+^ to Ti^4+^ imply the redox reaction 2Ti^3+^ + 2H^+^ → 2Ti^4+^ + H_2_ upon heating in inert atmospheres, resulting in the chemical composition Ti^IV+^P_2_O_7_ for Ti(IV)p.

Here, we now report on the ab initio structure determination of the two new pyrophosphate phases (Ti(III)p and Ti(IV)p) from the PXRD data. Especially, the structure of Ti(III)p as the only pyrophosphate phase with solely trivalent titanium ions in the bulk material is of special interest and, to the best of our knowledge, unique. Even though Ti(IV)p contains tetravalent titanium ions and pyrophosphate groups similar to the well-known cubic TiP_2_O_7_, the PXRD data indicate significant structural differences. To solve the structures of both phases, first, the local structure/coordination of both compounds was analyzed via spectroscopic and total scattering methods. With the knowledge of the average local structure, the average bulk crystal structure was derived. In addition, the reaction from Ti(III)p to Ti(IV)p via two endothermic processes was studied with *in situ* temperature-dependent (TD) PXRD and Raman spectroscopy.

## Experimental Section

### Material Synthesis

The Ti(III)p sample was prepared via the molten salt synthesis from a dried mixture of TiO_2_ (P25, Degussa, phase mixture of anatase and rutile, ≥99.5%) and NH_4_(H_2_PO_2_) (Fluka, ≥97.0%). The reaction mixture was heated to 573 K for 2 h in a protective N_2_ atmosphere. Ti(IV)p was obtained by annealing Ti(III)p under a protective atmosphere at 773 K for 4 h. The well-known cubic TiP_2_O_7_ was crystallized by heating Ti(III)p to 523 K in air. A detailed description of the synthesis approaches can be found in work by Stegmann et al.^26^

### PXRD

The PXRD experiments were performed on a STADI P diffractometer (STOE and Cie GmbH, Darmstadt, Germany) in transmission mode (*d*_capillary_ = 0.5 mm) using Cu Kα_1_ radiation. The instrument was equipped with a primary Ge(111) monochromator and a position-sensitive detector system. The diffraction patterns of Ti(III)p and Ti(IV)p were recorded with a step size of 0.01° 2θ and a measuring time of 30 and 60 s step^–1^, respectively. From Ti(III)p, the TD data (303–823 K) were collected on an X’Pert Pro diffractometer (Panalytical BV, Amelo, The Netherlands) equipped with a divergence slit (0.25°), an antiscatter slit (0.5°), a Soller slit (0.04 rad), and a mask (5 mm). The data were recorded with an X’Celerator Scientific detector system. Additionally, an XRK-900 reaction chamber (Anton Paar GmbH, Graz, Austria) was installed. The PXRD data were collected in a diffraction range of 10–36° 2θ with a step width of 0.0167 step/°. The sample was heated with 10 K min^–1^ to 823 K in an N_2_ atmosphere. The PXRD data were collected in 100 K steps in the temperature range from 100 to 300 K, in 5 K steps from 350 to 440 K, and in 50 K steps from 450 to 550 K. Hereafter, the sample was kept in synthetic air for 3 h. All PXRD patterns were analyzed with the *DiffracPlus Topas 6* software (Bruker AXS GmbH, Karlsruhe, Germany).^27^

### Total Scattering Experiments and Subsequent Pair Distribution Function (PDF) Analysis

The data for the total scattering experiment and subsequent PDF analysis were collected at Petra III (Beamline P02.1, DESY, Hamburg, Germany) using a wavelength of 0.20709 Å. For data collection, a Varex XRD 4343DT detector (150 × 150 μm^2^ pixel size; 2880 × 2880 pixel area) was used. The PDFs were generated with the *PDFgetX3* software (Columbia University, New York, NY).^28^ The local structure refinement of the PDF data was performed with *PDFgui*.^29^*Q*_damp_ (0.0304 Å^–1^) and *Q*_broad_ (0.00253 Å^–1^) were determined with a silicon standard.

### Solid-State NMR Spectroscopy

The ^31^P magic-angle-spinning (MAS) NMR spectra were recorded on a Bruker Avance III HD 500WB spectrometer (Bruker BioSpin GmbH, Rheinstetten, Germany) using a double-bearing MAS probe (DVT BL4) at a resonance frequency of 202.5 MHz. The spectra were measured by applying single π/2 pulses (3.0 μs) with a recycle delay of 600 s (eight scans) at several spinning rates between 3 and 10 kHz. A high-power proton decoupling (spinal64) was applied. The chemical shifts are given with respect to 85% aqueous H_3_PO_4_ using solid NH_4_H_2_PO_4_ as a secondary reference [δ_iso_(NH_4_H_2_PO_4_) = 0.81 ppm].^30^ The spectral simulations were performed using the solids line-shape analysis module implemented in the *TopSpin 3.2* NMR software package from Bruker BioSpin GmbH. For conversion of the screening data calculated by CASTEP into chemical shift data, the following relationship was used: δ_iso_(P_*n*_) = σ_iso_(NH_4_H_2_PO_4_) + δ_iso_(NH_4_H_2_PO_4_) – σ_ii_(P_*n*_) = 285.79 ppm – σ_ii_(P_*n*_). For σ_iso_(NH_4_H_2_PO_4_), CASTEP yields 284.98 ppm. The isotropic chemical shift δ_iso_(NH_4_H_2_PO_4_) with respect to 85% aqueous H_3_PO_4_ is 0.81 ppm.^30^ The span Ω and skew κ are defined in the usual way: Ω = σ_33_ – σ_11_ = δ_11_ – δ_33_ and κ = 3(σ_iso_ – σ_22_)/Ω = 3(δ_22_ – δ_iso_)/Ω with σ_11_ ≤ σ_22_ ≤ σ_33_ and δ_11_ ≥ δ_22_ ≥ δ_33_.^31^

### Raman Spectroscopy

The Raman data were recorded with an InVia spectrometer (Renishaw Ltd., Wotton-under-Edge, U.K.) using an excitation wavelength of 785 nm; the laser power was tuned to 30 mW. A 1200 grating mm^–1^ grid assured a spectral resolution of 1 cm^–1^. All spectra were collected with 10 s step^–1^ and three repetitions. Additionally, a TD measurement of Ti(III)p was performed in a CCR reaction cell (Linkam scientific instruments, Epsom, U.K.). The sample was heated to 923 K at 10 K min^–1^ in a N_2_ atmosphere.

### Density Functional Theory (DFT) Calculation

DFT calculations for Ti(III)p and Ti(IV)p were carried out with the CASTEP code, version 17.^32^ All calculations used the Perdew–Burke–Ernzerhof (PBE) exchange-correlation functional with the pairwise dispersion correction devised by Tkatchenko and Scheffler.^33,34^ In each case, the coordinates of all atoms were optimized, fixing the unit cell parameters to experimental values. Spin-polarized calculations were performed for Ti(III)p. These calculations used on-the-fly-generated (OTFG) ultrasoft pseudopotentials and a cutoff energy of 750 eV for the plane-wave basis set. The first Brillouin zone was sampled using a 3 × 2 × 3 *k* mesh, corresponding to five irreducible *k* points. Because CASTEP does not support linear response calculations for systems with unpaired electrons, vibrational frequencies were calculated using the finite displacement method. Because of this methodological limitation, no Raman intensities could be predicted for Ti(III)p. DFT calculations for Ti(IV)p used OTFG norm-conserving pseudopotentials and a cutoff energy of 1200 eV. The first Brillouin zone was sampled using a 3 × 2 × 3 *k* mesh (nine irreducible *k* points). For this system, the vibrational calculation made use of the linear response method, enabling the prediction of Raman intensities.^35−37^ The calculation of ^31^P NMR shifts for Ti(IV)p employed the gauge-including projector-augmented-wave method implemented in CASTEP, using OTFG ultrasoft pseudopotentials with a cutoff energy of 871 eV.^38,39^ The reference calculation for NH_4_H_2_PO_4_ was carried out based on a fully ordered structure model of this compound proposed by Baur (space group *P*2_1_2_1_2_1_), again optimizing all atomic coordinates.^40^

### Electrochemical Impedance Spectroscopy (EIS)

The EIS measurements of Ti(III)p and Ti(IV)p were performed with an IM6 Zahner (Messsysteme) impedance spectrum analyzer under hydrated [samples under deionized (DI) water] and anhydrous conditions (N_2_ atmosphere). For this purpose, powders of Ti(III)p and Ti(IV)p were pelleted at a compression force of 14 ton cm^–2^ and clamped between golden stainless steel electrodes in a two-electrode cell. The EIS spectra were recorded at a direct-current voltage of 0 mV and a sinusoidal voltage perturbation of 100 mV in the frequency range of 4 MHz to 10 Hz. The proton conductivities were detected by fitting the half-circles in the Nyquist spectra.

## Results

### Average Local Structure Analysis

The investigation of the chemical compositions of Ti(III)p and Ti(IV)p results in the stoichiometric formulas NH_4_TiP_2_O_7_ and TiP_2_O_7_.^26^ The XPS spectra of Ti(III)p indicate tri- and tetravalent titanium species, while for Ti(IV)p, only tetravalent titanium species were found. For the structure determination, the average local structure was determined via Raman spectroscopy as well as complementary PDF data analysis. The obtained information about the polyhedral coordination was used for the ab initio crystal structure solution of both structures from PXRD.

**Figure 2:**
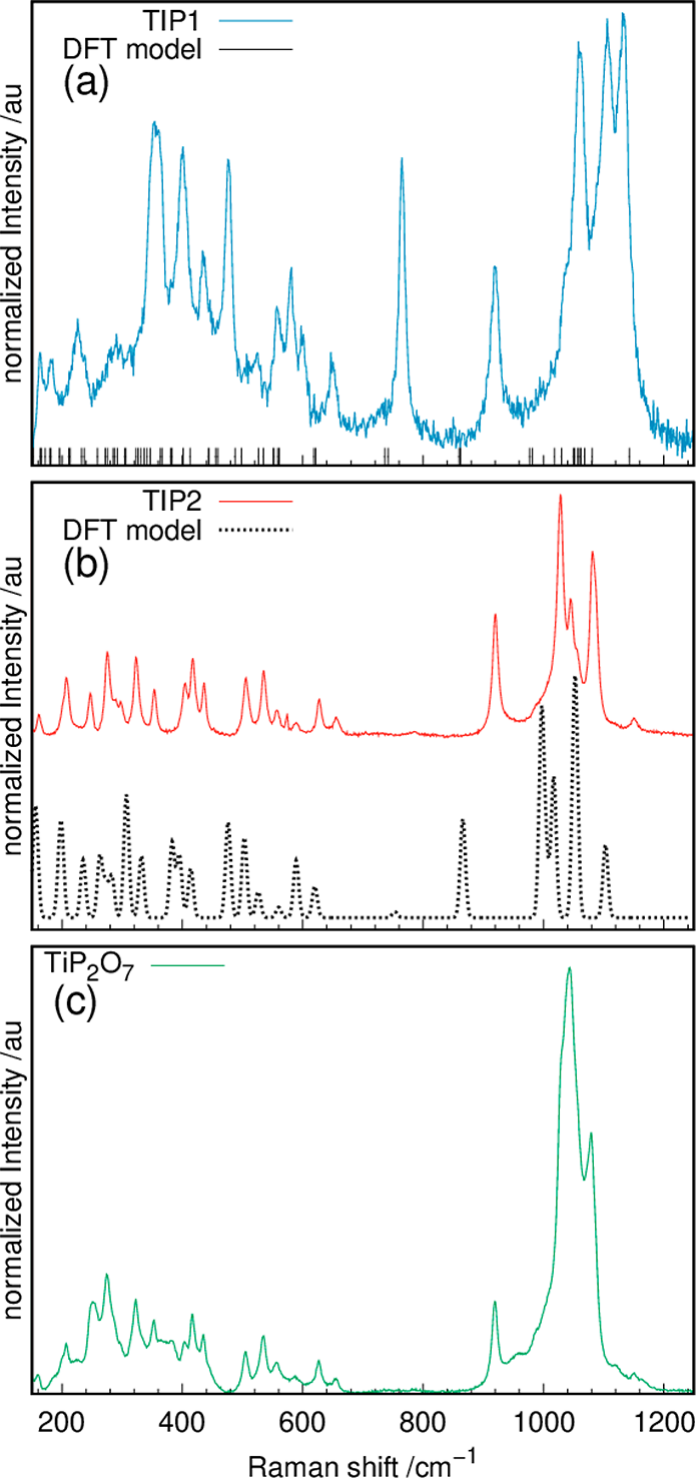
Measured Raman spectra of (a) Ti(III)p and (b) Ti(IV)p, together with their theoretical Raman shifts (Ti(III)p) or spectra (Ti(IV)p) obtained from DFT modeling, as well as (c) the Raman spectrum of TiP_2_O_7_.

Spectroscopic methods like Raman spectroscopy enable analysis of the coordination polyhedra. The Raman spectrum of Ti(III)p (Figure 2a) shows the symmetric stretching vibration of PO_*x*_ polyhedra [ν_s_(P–O)] in the range from ∼900 to 1200 cm^–1^ and the P–O–P deformation vibration at 920 cm^–1^.^25,41,42^ In particular, stretching modes show a high correlation of the Raman shift with the bond length, which itself depends heavily on the second coordination sphere of PO_*x*_ polyhedra.^25^ This allows orthophosphates [*r*(P–O) = 150–185 pm; ν_s_(P–O) = 900–1100 cm^–1^], pyrophosphates [*r*(P–O) = 1.45–1.56 Å; ν_s_(P–O) = 975–1250 cm^–1^], or metaphosphates [*r*(P–O) = 1.45–1.54 Å; ν_s_(P–O) = 1050–1150 cm^–1^] to be assigned.^25^ The symmetrical stretching vibration ν_s_(P–O) of Ti(III)p [1035(1)–1135(1) cm^–1^] fits well to both pyro- and metaphosphates. The spectrum of Ti(III)p shows also the P–O–P symmetric stretching vibration [ν_s_(P–O–P)] at 765(1) cm^–1^, together with the P–O–P deformation vibration [δ(P–O–P) = 920(1) cm^–1^], which are both characteristic for bent pyro- and metaphosphates.^25,41^ Also, tetrahedrally coordinated TiO_4_ polyhedra and Ti–O–Ti chains show a stretching mode at ∼750 cm^–1^, but because of the appearance of characteristic TiO_6_ octahedral modes at 399, 519, and 639 cm^–1^, TiO_4_ and Ti–O–Ti chains are regarded as unlikely.^41−43^ The broadness of the TiO_6_ modes implies highly distorted octahedra. In the spectral range from ∼370 to 650  cm^–1^, additional P–O modes (O–P–O deformation and PO_4_^3–^ bending modes) are observed.

The Raman spectrum obtained for Ti(IV)p (Figure 2b) shows mainly the same modes as those described for Ti(III)p. The symmetric stretching vibrations shift to smaller wavenumbers [ν_s_(P–O) = 1012(1)–1150(1) cm^–1^]. The P–O–P deformation vibration [δ(P–O–P)] shows a comparable intensity, but the intensity of the symmetric stretching vibration at ∼750 cm^–1^ is lower (compare Figure 2a). The well-known TiP_2_O_7_ crystallizing in a superstructure with *Pa*3̅ symmetry displays no symmetrical P–O–P stretching mode (Figure 2c).^24^ TiP_2_O_7_ consists of linear and bent pyrophosphates, implying a change in the P–O–P angle for Ti(IV)p. Additionally, all but the TiO_6_ octahedral modes in the spectral range from ∼370 to 650 cm^–1^ are more defined (compare Figure 2a) and show higher comparability to the spectrum of the well-ordered TiP_2_O_7_ (compare Figure 2c), indicating a higher degree of symmetry of the coordination polyhedra.

**Figure 3:**
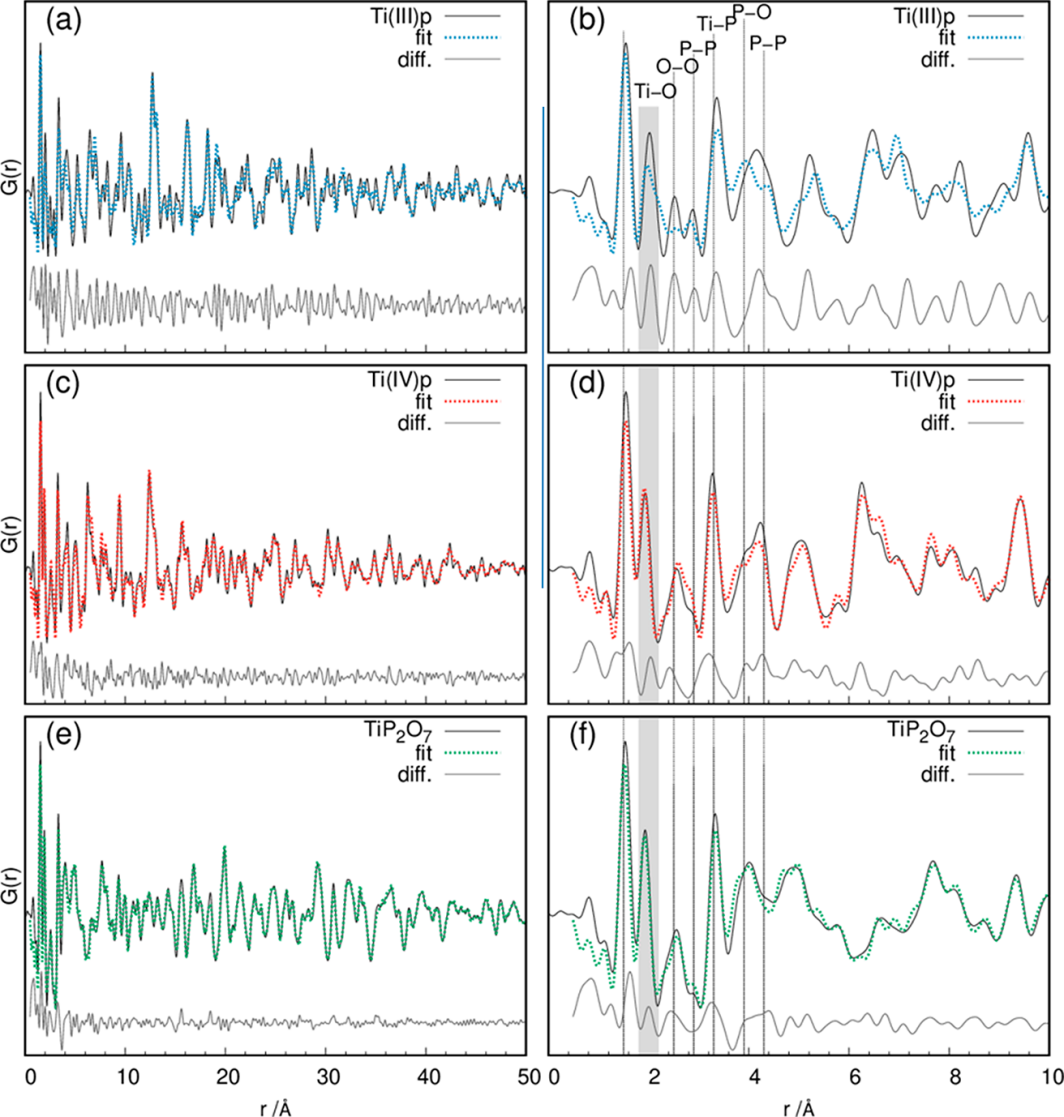
Experimental and fitted PDF data of (a and b) Ti(III)p, (c and d) Ti(IV)p, and (e and f) TiP_2_O_7_ and the respective difference curves (gray). In parts b, d, and f, the corresponding atom pairs are marked with lines.

Total scattering experiments and subsequent PDF analysis (Figure 3) of all three samples were performed to investigate their average local structures. Therefore, the PDF data were at first analyzed qualitatively for a comparison of the data. A subsequent refinement based on the solved crystal structure models will be discussed during validation of the respective structure models.

The experimental PDF of Ti(III)p (Figure 3a,b) shows atom pair correlations at 1.544(1) Å and 252.2(2) pm fitting well to the known P–O [*r*(P–O) = 1.45–1.58 Å] and O–O [*r*(O–O) = 2.48(2) Å] distances in the [P_2_O_7_]^4–^ group.^25,44^ The Ti–O pair correlation at 2.026(1) Å is remarkable, which is significantly elongated in comparison to the typical bond distances of Ti^IV+^–O in titanium phosphates, which vary between 1.885(1) and 1.945(1) Å.^23,24^ The observed Ti–O pair correlation of Ti(III)p [*r*(Ti–O) = 2.026(1) Å] is in good agreement with the Ti^III+^–O distances [*r*(Ti^III+^–O) = 2.033(2)–2.029(2) Å] in Ti(PO_3_)_3_).^18^ Thus, the PDF data are clear proof for the incorporation of Ti^3+^ in the crystal structure of Ti(III)p. The Ti^4+^ species observed by surface-sensitive XPS is solely located on the crystal surfaces, while the bulk structure contains Ti^3+^.^26^ The P–P distances at 2.881(2) Å correlate well to pyrophosphates [TiP_2_O_7_: *r*(P–P) = 2.99 Å], which is also in agreement with the Raman results (Figure 2a).^24^ Additional pair correlations from the secondary coordination sphere in the range from 3.92 to 4.46 Å can be correlated to the P–O (3.501–4.776 Å) and P–P (4.103–4.786 Å) distances.^24^ The PDF analysis implies that the Ti(III)p structure consists of pyrophosphates connected by Ti^III+^O_6_ polyhedra.

The comparison of the PDFs of Ti(III)p (Figure 3a,b) and Ti(IV)p (Figure 3c,d) shows the presence of similar coordination polyhedra. After the reaction of Ti(III)p to Ti(IV)p, the P–O and P–P pair correlations remain almost unchanged at 1.5494(9) and 2.880(4) Å (Figure 3c,d). In addition, the O–O distances remain quite similar [Ti(IV)p, 2.557(3) Å; Ti(III)p, 2.522(2) Å]. This indicates that the pyrophosphate unit remains stable during the reaction. The major differences are observed for the Ti–O pair correlation, which shortens significantly to 1.933(1) Å. Also, the Ti–P pair correlation shifts to smaller distances [3.2756(9) Å], mirroring the oxidation of Ti^3+^ to Ti^4+^ during the reaction of Ti(III)p to Ti(IV)p.^24^

The experimental PDFs of Ti(III)p (Figure 3a,b) and Ti(IV)p (Figure 3c,d) show both similarities to the PDF of TiP_2_O_7_ (Figure 3e,f) for short pair correlations. The pair correlations belonging to the first and second coordination spheres around the metal atoms up to ∼4.50 Å illustrate the structural relationship of the three compounds. However, the differences appearing at longer distances indicate major differences in the long-range order.

Analysis of the experimental PDF data reveals the incorporation of trivalent titanium species in the bulk structure of Ti(III)p. Further, the average local structure consists of TiO_6_ octahedra and pyrophosphates [P_2_O_7_]^4^. Ti(IV)p, on the other hand, consists of tetravalent titanium ions. Analysis of the spectroscopic and scattering data reveals intact TiO_6_ octahedra and pyrophosphate [P_2_O_7_]^4–^ units. The Raman spectra indicate a more regular coordination polyhedron.

### Average Bulk Structure Determination of the Ti(III)p Structure

Indexing of the PXRD data of Ti(III)p results in a monoclinic unit cell with the metric parameters *a* = 7.5539 Å, *b* = 10.2642 Å, *c* = 8.2657 Å, and β = 105.86° (goodness of fit = 616.46). A Pawley fit of the measured data in space group *P*2_1_/*c*, with the refined metric parameters summarized in Table 1, shows the best agreement.

**Table 1:** Metric Parameters Obtained from Pawley Fitting

Ti(III)p: *P*2_1_/*c* (*R*_wp_ = 6.35%)^a^		Ti(IV)p: *P*1̅ (*R*_wp_ = 5.91%)^a^	
*a* (Å)	7.5457(2)	*a* (Å)	6.2287(1)
*b* (Å)	10.2550(2)	*b* (Å)	7.9489(1)
*c* (Å)	8.2573(6)	*c* (Å)	6.2063(1)
		α (deg)	102.807(2)
β (deg)	105.925(6)	β (deg)	74.817(2)
		γ (deg)	83.196(2)

a *R*_wp_: weighted-profile *R* factor.

The structure of Ti(III)p was determined via *simulated annealing* (ab initio structure solution). For the input file, the information about the unit cell metric and the obtained chemical composition (NH_4_^+^, P_2_O_7_^4–^, and Ti^3+^) was combined. For the pyrophosphate and ammonium groups, rigid bodies (Figure S1) were constructed. In the case of pyrophosphates, the rotational degree of freedom of the P–O–P bonds was taken into account.

**Figure 4:**
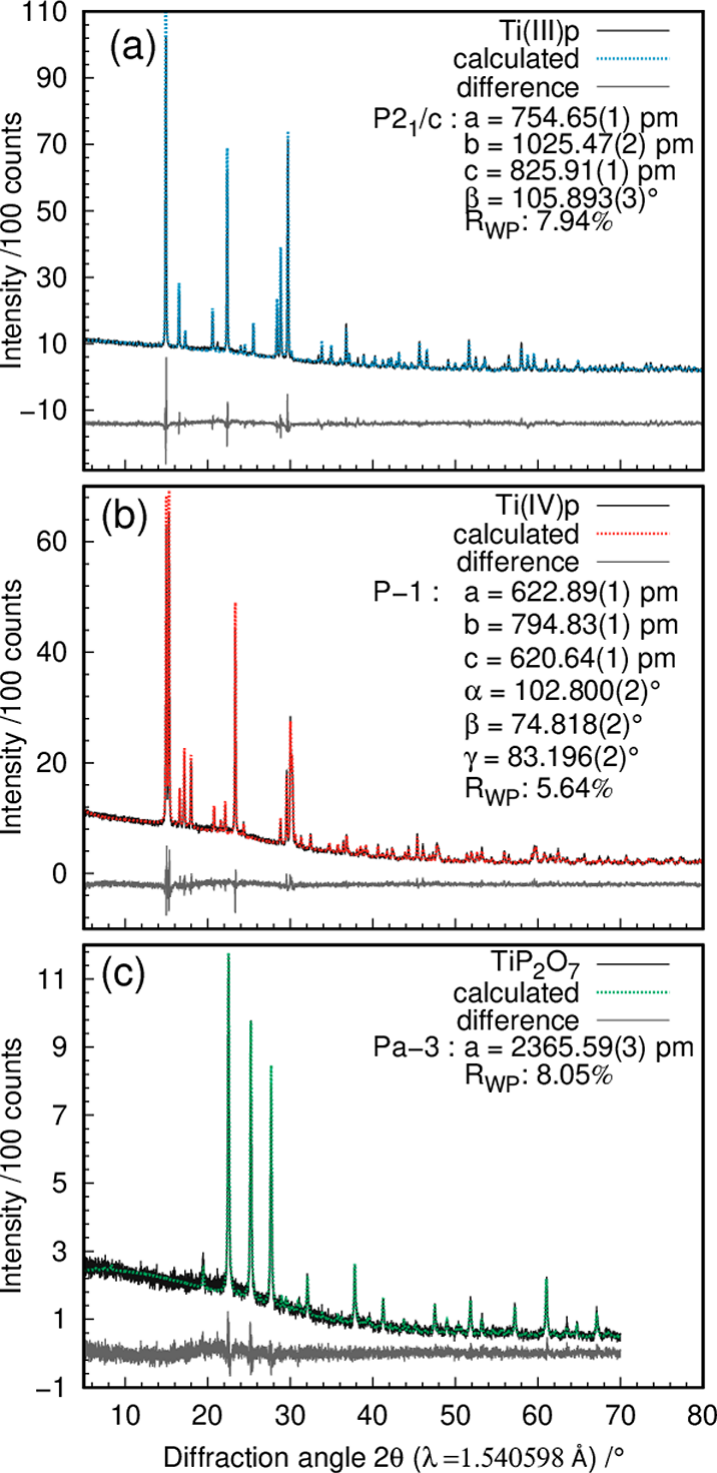
Rietveld refinement plots for (a) Ti(III)p, (b) Ti(IV)p, and (c) TiP_2_O_7_. The measured PXRD data are displayed as solid lines, the calculated PXRD patterns from the refined models are shown as dotted lines, and the difference curves are shown as solid gray lines.

**Figure 5:**
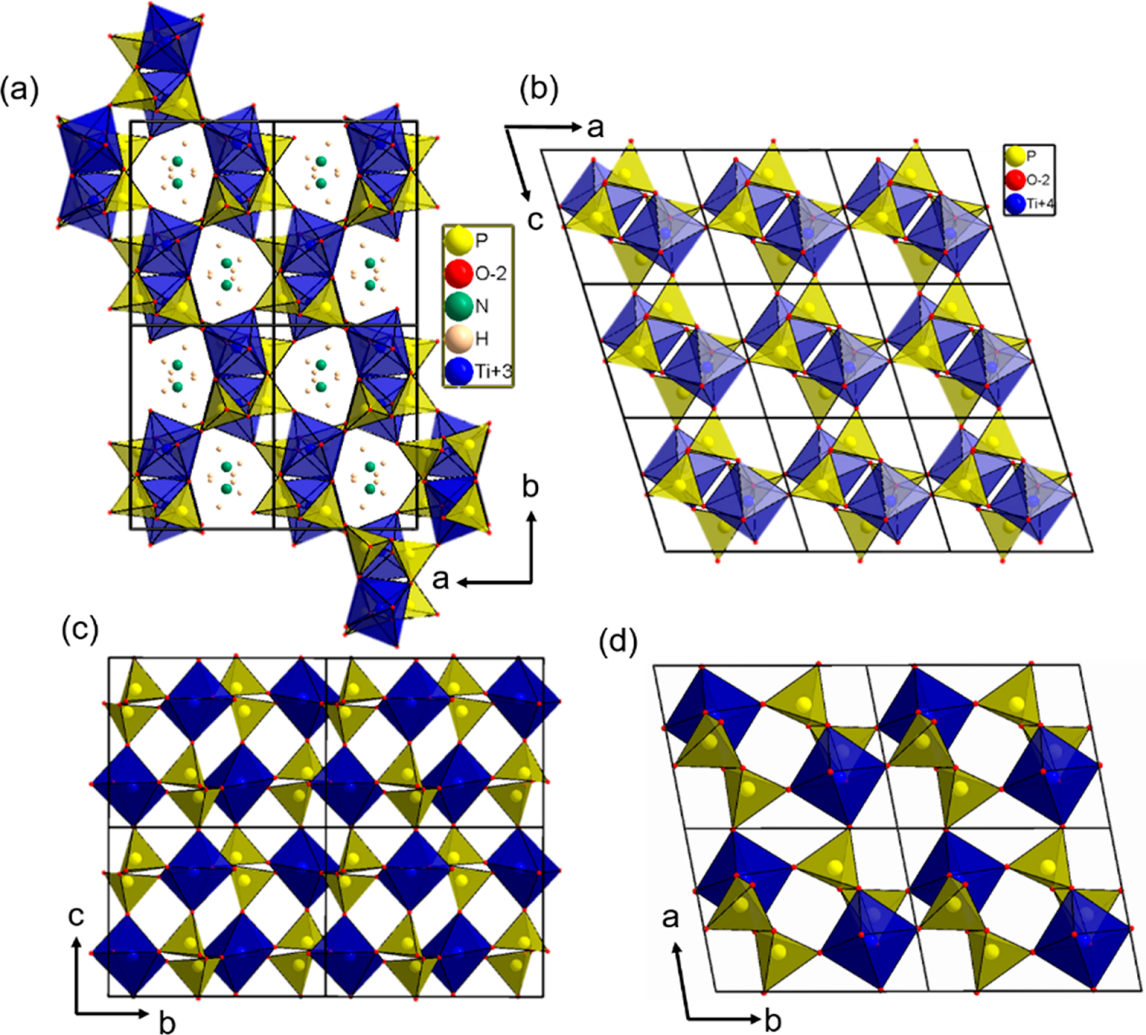
(a) Crystal structure of Ti(III)p viewed along the one-dimensional channel with incorporated NH_4_^+^ ions. (b) Crystal structure of Ti(IV)p displaying the empty one-dimensional channels. (c) [TiP_4_O_12_] layers of the Ti(III)p structure in the *ab* plane, For the sake of clarity, the NH_4_^+^ ions are not included. (d) [TiP_4_O_12_] layers in the Ti(IV)p structure displayed in the *ab* plane.

The resulting structure models obtained by the *simulated annealing* approach were subsequently refined with the Rietveld method (Figure 4a). The refinement shows a good agreement between the model and the measured data with a residual value *R*_wp_ of 7.94%. The crystal structure of Ti(III)p (Figure 5a,b) consists of TiO_6_ octahedra connected via corner-sharing [P_2_O_7_]^4–^ polyhedra. Each TiO_6_ octahedron is coordinated by five [P_2_O_7_]^4–^ groups: four as single-side ligands and one as a bidentate ligand. This is different from that in the well-known cubic TiP_2_O_7_ structure, where every [P_2_O_7_]^4–^ group is connected to the tetravalent titanium via an individually coordinating bond, resulting in an arrangement of [P_2_O_7_]^4–^ and TiO_6_, which can be related to the NaCl-type structure.^23,24^ Ti(III)p consists of two [TiP_4_O_12_] layers (Figure 5c), translated relative to each other. The three-dimensional arrangement of these layers results in one-dimensional channels [*d*_min_ = 2.46(1) Å; *d*_max_ = 3.93(1) Å] running along the crystallographic *c* axis (Figure 5a). The channels are stabilized by NH_4_^+^ ions coordinating the negatively charged [TiP_2_O_7_]^−^ framework (Figure 5a). The calculation of the Fourier difference map (Figure S2) shows a residual electron density surrounding the incorporated NH_4_^+^ ions. This can be explained by the dynamical disorder of NH_4_^+^ and/or stacking faults in the structure resulting in partial blocking of the channels. The Ti(III)p structure can be related to the high-voltage pyrophosphate cathode material Li_2_FeP_2_O_7_ also crystallizing in *P*2_1_/*c* [*a* = 11.01589(7) Å, *b* = 9.75416(6) Å, *c* = 9.80462(6) Å, and β = 101.5444(6)°].^9,45,46^ Li_2_FeP_2_O_7_ consists of a three-dimensional arrangement of undulating [Fe_4_P_8_O_32_]_∞_ layers building a channel system that is occupied by Li^+^ ions.^9,45,46^ As such, the channel structure of Ti(III)p may prove appropriate for ion conductivity, provided the framework structure is stable when NH_4_^+^ is exchanged with other cations.

A more detailed discussion of the structural parameters of the two materials is provided in the Supporting Information.

To validate the structure model determined by *simulated annealing* and subsequent Rietveld refinement, a refinement of the PDF data based on the determined structure model from *simulated annealing* and subsequent Rietveld refinement was performed. For the fitting procedure, the scaling factor, spherical shape correction factor, Debye–Waller factors for each atomic species, lattice parameters, and δ_1_ values were considered for atom pair correlations from 1 to 50 Å. The fit of the data obtained for Ti(III)p (Figure 3a) is in good agreement with the measured data. Especially, the experimentally derived distances in the first two coordination spheres fit well to both the literature values of similar systems and the solved crystal structure.^23,24^ For longer distances, the deviation between the model and measured data increases. This mismatch can be explained by a partial collapse of the channel structure, which may result from the stacking faults of the [TiP_4_O_12_] layers (Figure 5b) or the local differences in the NH_4_^+^ positions and occupancy (see the Supporting Information for details).

Besides, the active Raman vibrations of the proposed models with their corresponding Raman shifts were calculated by DFT methods. Because of the presence of the trivalent titanium ion in Ti(III)p with its unpaired electron spin, no intensities could be calculated for the modes of that material. Therefore, only the Raman shifts of the modes are marked in Figure 2a. The calculated Raman modes are shifted to lower wavenumbers, a phenomenon that is often observed for DFT calculation using the PBE functional.^47^ Apart from that, the measured Raman spectrum and the calculated positions of the Raman active modes are in good agreement. All observed Raman modes can be correlated to the calculated shifts. The additional theoretical positions may not be detectable in the experimental data because of low intensities. Thus, the fitting of the total scattering experiments as well as the high comparability of the measured and theoretical spectra derived from DFT calculations corroborate the successful structure solution of the Ti(III)p phase.

### Average Bulk Structure Determination of the Ti(IV)p Structure

The indexing of the PXRD data of the Ti(IV)p phase resulted in a triclinic unit cell in space group *P*1̅ (goodness of fit = 46.05). The lattice parameters derived by subsequent Pawley fitting are listed in Table 1. For structure determination of Ti(IV)p via a *simulated annealing* technique, the unit cell information and a rigid body of the [P_2_O_7_]^4–^ group (Figure S1) were combined. The subsequent Rietveld refinement shows good agreement of the calculated and measured data with an *R*_wp_ value of 5.64% (Figure 4b). The resulting structure model (Figure 5b,d) reveals that the [TiP_4_O_12_] layers (Figure 5d) and the channel system [*d*_min_ = 2.01(1) Å; *d*_max_ = 5.07(1) Å; Figure 5b] are maintained during the release of NH_3_ and H_2_, which go along with the oxidation of Ti^III^ to Ti^IV^. Calculation of the Fourier difference map (Figure S2) shows a residual electron density in the channel system, which might result from water adsorbed from ambient air.

The geometric structure parameters of Ti(IV)p are summarized and discussed in detail in Tables S2 and S4.

To also validate the determined structure of Ti(IV)p, the structure model was refined against the respective PDF data (Figure 3c,d). The refinement shows a good match at large distances (*R*_w_ = 28.4246). The PDF calculated from the refined structure model with elongated bridging P–O distances [*r*(P–O1) = 1.60(1) Å] and somewhat shorter terminal P–O distances [*r*(P–O2–O7) = 1.54(4) Å] shows good agreement with the PDF of the measured data. Besides, the intramolecular P–P [2.83(1) Å] and O–O (between ∼2.40 and 2.50 Å) distances fit well to the described model. Moreover, also the Ti–O distances in the TiO_6_ octahedra fit well with the model. The improved fit in the higher *r* range indicates a higher long-range order displaying structural relaxation during the release of NH_3_ and H_2_ and oxidation of the titanium, resulting in a more regular channel structure (Figure 5).

Further, the structural model was validated by spectroscopy. Figure 2b displays the calculated Raman spectrum including theoretical intensities. Both the calculated intensities and the positions fit well to the experimental spectrum (Figure 2b). The biggest deviation between the calculated and measured data is the shift of the deformation mode of the pyrophosphate [δ(P–O–P) = 920(1) cm^–1^].^41^ This mode highly depends on the P–O–P angle. Determination of the latter via PXRD underlies systematic errors. A noteworthy similarity is the comparable intensity of the observed and calculated symmetrical stretching vibrations ν_s_(P–O–P) [765(1) cm^–1^], indicating the change in the P–O–P angle as described earlier.^25,41,42^ Similar to the Ti(III)p model, a shift of the calculated Raman modes of Ti(IV)p to lower wavenumbers is observed, a phenomenon that is commonly observed for DFT calculation using the PBE functional.^47^

**Figure 6:**
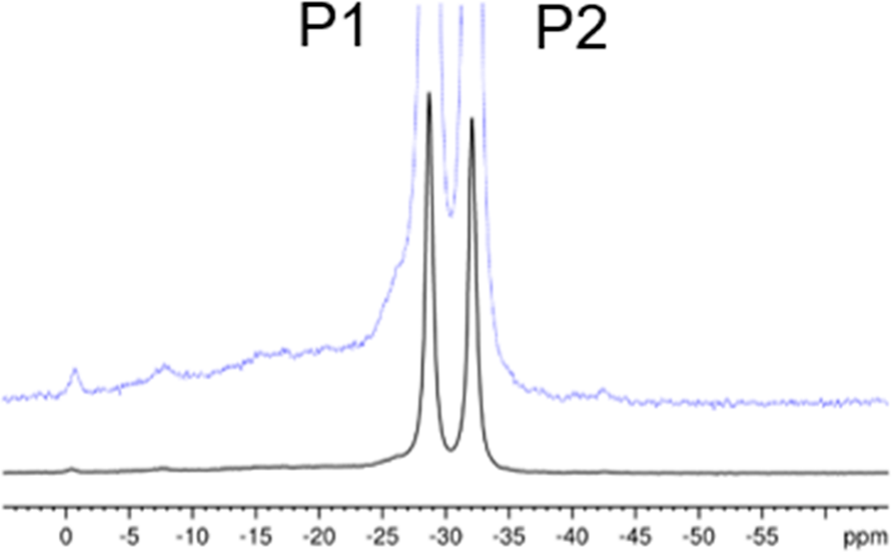
^31^P MAS NMR spectrum of the same Ti(IV)p sample that was used for the PXRD measurement (ν_MAS_ = 10 kHz). The dashed blue line depicts the same spectrum magnified by a factor of 8. The width of both resonance lines (fwhh) is about 140 Hz. The assignment is based on the results of DFT calculations.

**Figure 7:**
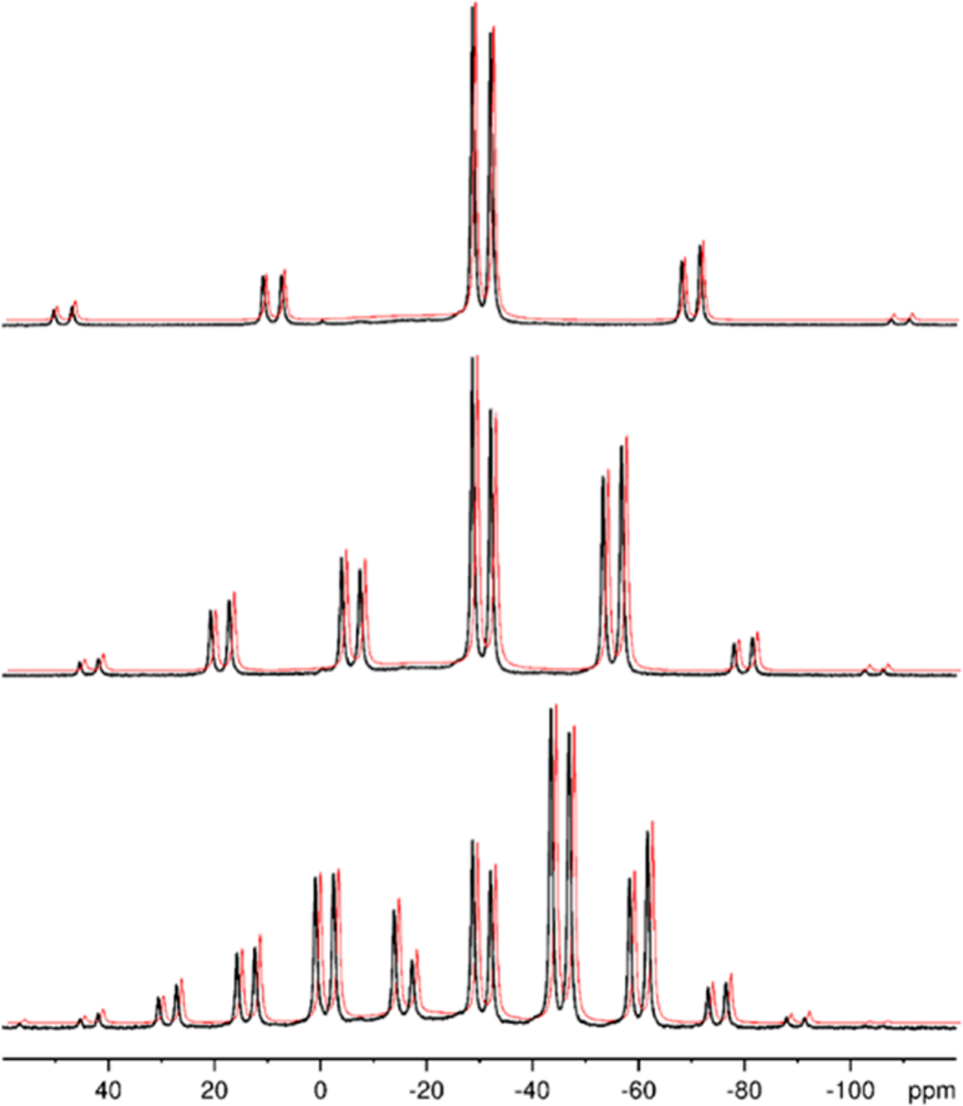
^31^P MAS NMR spectra of Ti(IV)p measured at different spinning speeds. The data were shifted for the sake of clarity. The experimental spectra (black curves) are shown in comparison with those calculated using the parameters given in Table 2 for ν_MAS_ = 5 kHz (red curves).

Finally, support for the validity of the structural solution found for Ti(IV)p comes from ^31^P NMR. NMR spectroscopy is very sensitive to the local geometry around the nucleus studied. Figure 6 shows that the two inequivalent phosphorus sites in the pyrophosphate units give rise to well-resolved resonance lines at −28.7 and −32.1 ppm. The line widths (full width at half-height, fwhh) for both resonance lines measured varied between 120 and 150 Hz. The additional shoulder at about −26 ppm and the other broad lines at the low-field side are assigned to crystal defects and amorphous byproducts. The relative intensities of these additional contributions varied from sample to sample, with a total intensity in the range of 5–12% of the phosphorus detected. Not only the isotropic chemical shift but also its anisotropy contain information about the local geometry around the nucleus. The two parameters describing the chemical shift anisotropy, the span Ω and skew κ, can be extracted from the MAS NMR spectra by a well-established procedure.^31,48^ To determine these parameters for the two phosphorus atoms in Ti(IV)p, we used spectra taken at three different spinning speeds (Figure 7). The thus-obtained data are reported in Table 2 together with the results of DFT calculations (CASTEP).

**Table 2:** Chemical Shift Data for Ti(IV)p as Obtained by ^31^P MAS NMR and DFT Calculations

		δ_iso_ (ppm)		Ω (ppm)		κ	
	ν_MAS_ (kHz)	P1	P2	P1	P2	P1	P2
MAS NMR	3	–28.7	–32.1	101.4	110.0	–0.45	–0.63
	5	–28.7	–32.1	101.3	110.3	–0.45	–0.63
	8	–28.7	–32.1	104.4	111.1	–0.39	–0.62
CASTEP		–27.1	–31.3	100.7	110.3	–0.54	–0.89

We regard the data derived from the spectra measured at 3 and 5 kHz as the most reliable ones. In general, the highest reliability for the determination of shift tensor components from MAS NMR spectra is achieved when the central band is surrounded by five to seven spinning sidebands of significant intensities.^49^ Furthermore, at low spinning speeds, any thermal effects caused by frictional heating can be neglected.^50^

From the data given in Table 2, the assignment of the two resonance lines in Figure 6 is obvious. The differences between the calculated and experimental isotropic chemical shifts fall into the range obtained for other phosphates.^51^ Not only the isotropic chemical shifts but also the spans Ω are nicely reproduced by the DFT calculations. The low-field line has a considerably smaller span than the high-field one, in both theory and experiment. The only significant differences occur for the values of the skew κ, i.e., of the parameter that describes the position of δ_22_ (σ_22_) with respect to δ_11_ (σ_11_) and δ_33_ (σ_33_). Similarly good agreement with the experiment in the span values and comparably large deviations in the skew values have been observed in previous DFT investigations on inorganic phosphates, which used an analogous computational approach.^52,53^

Because the DFT calculations are based on the structural data, the rather good agreement between the experimental and calculated chemical shift data delivers sound evidence for the validity of the structure solution.

Both novel titanium phosphate structures belong to the class of transition-metal pyrophosphates, MP_2_O_7_. Most MP_2_O_7_ compounds (M = Si, Sn, Pb, Ti, Zr, Hf, U, Ce) crystallize in a 3 × 3 × 3 superstructure in the space group *Pa*3̅.^5,23,24^ In MP_2_O_7_ structures, all TiO_6_ octahedra are connected via corner-sharing oxygen atoms to six [P_2_O_7_]^4–^ groups. Hereby, the pyrophosphates and TiO_6_ octahedra are arranged in a loosely NaCl-type structure.^23^ The usage of NH_4_(H_2_PO_2_) in the synthesis causes not only the stabilization of titanium in the oxidation state III+ but also the incorporation of NH_4_^+^ ions in the channels of the structure.^26^ Both the change of the oxidation state and the incorporation of NH_4_^+^ ions in Ti(III)p cause a change in the arrangement of the TiO_6_ octahedra and [P_2_O_7_]^4–^ groups. Unlike in the cubic TiP_2_O_7_ structure, five [P_2_O_7_]^4–^ groups are connected via corner-sharing oxygen atoms and one [P_2_O_7_]^4–^ group is connected via two oxygen atoms. This arrangement causes the formation of big one-dimensional channels stabilized by the NH_4_^+^ ions.

Heating Ti(III)p causes the thermal decomposition of NH_4_^+^ and the oxidation of Ti^III^ to Ti^IV^. In air, Ti(III)p reacts to the well-known TiP_2_O_7_. As described above, the reaction includes major changes in the Ti–P network. Heating of Ti(III)p in inert atmospheres, on the other hand, results in the formation of Ti(IV)p. Despite the fact that this reaction is also driven by the decomposition of NH_4_^+^ and the oxidation of titanium, the Ti–P network of Ti(III)p remains stable.

### Proton Conductivities of Ti(III)p and Ti(IV)p

**Figure 8:**
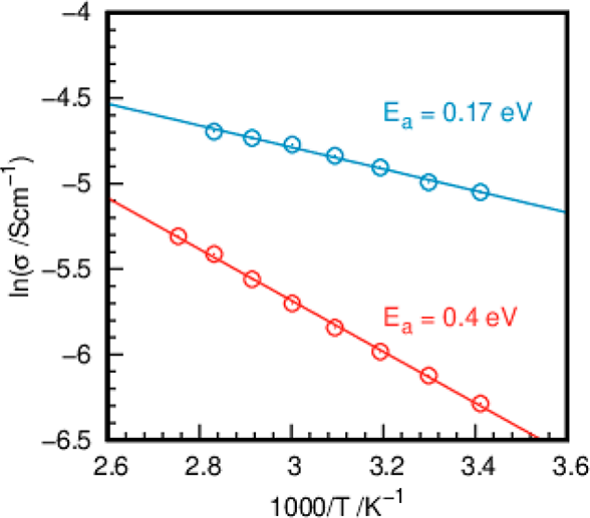
Arrhenius plots of the conductivities of Ti(III)p (blue) and Ti(IV)p (red) as a function of the temperature under fully hydrated (under DI water) conditions.

As mentioned above, Ti(III)p shows a structure related to the known cathode material Li_2_FeP_2_O_7_. Both consist of a channel system stabilized by incorporated ions. Consequently, we studied the proton conductivities of Ti(III)p and Ti(IV)p. Impedance spectroscopy on Ti(III)p and Ti(IV)p was performed under hydrated and anhydrous conditions. While the anhydrous samples do not allow an efficient proton migration, showing conductivities in the range of 10^–6^ S cm^–1^, the presence of liquid water increases the conductivities by 3 orders of magnitude (Figure S5) to the range of 10^–3^ S cm^–1^. The high proton conductivities under fully hydrated conditions (samples immersed in DI water) were accompanied by low activation energies for the proton transport of 0.17 and 0.4 eV for Ti(III)p and Ti(IV)p, respectively, as shown by the Arrhenius plots in Figure 8. The specific proton conductivity and the activation energy of Ti(III)p belong to the highest and lowest, respectively, ever reported for this class of materials and indicate charge transport based on the Grotthus mechanism, where proton migration is mediated by the formation of hydronium ions. Differences in the conductivity and activation energy between Ti(III)p and Ti(IV)p are assumed to correlate with the presence of ammonium ions in the channels of Ti(III)p, where they might act as proton donors and promote proton migration.

### Reactions in the Ti(III)p, Ti(IV)p, and TiP_2_O_7_ System

From *ex situ* annealing experiments, it is known that Ti(III)p with titanium in the oxidation state III+ transforms via the triclinic Ti(IV)p to the cubic TiP_2_O_7_ structure with both titanium ions in the oxidation state IV+.^26^ To investigate the structural relationship of Ti(III)p, Ti(IV)p, and cubic TiP_2_O_7_, *in situ* TD PXRD experiments were performed.

**Figure 9:**
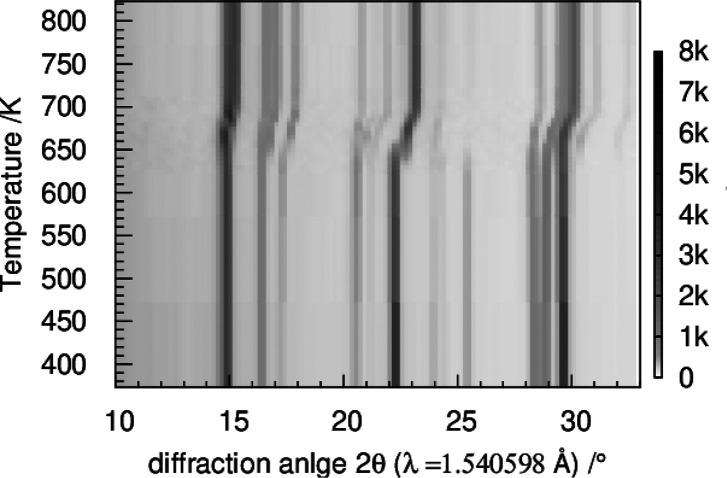
PXRD data obtained during heating of Ti(III)p in an inert atmosphere.

Ti(III)p was heated first to 823 K under an inert atmosphere to study the phase transition Ti(III)p → Ti(IV)p. In a second step, the atmosphere was switched to synthetic air and the sample was kept at 823 K for 180 min. The reflections of monoclinic Ti(III)p remain unchanged up to 633 K (Figure 9). In the temperature range from 638 to 658 K, the reflections shift to higher diffraction angles, indicating a negative thermal expansion. This contraction of the unit cell correlates with thermal decomposition of the incorporated NH_4_^+^ ions to NH_3_ and H^+^. The TGA–MS results imply a successive reduction of the formed H^+^ ions to H_2_.^26^ This reaction goes along with the oxidation of titanium during the reaction from Ti(III)p to Ti(IV)p as reported above. Thus, a redox reaction between the formed H^+^ and Ti^3+^ (2H^+^ + 2Ti^3+^ → H_2_ + 2Ti^4+^) proceeds during the transformation. Simultaneous with the release of the gaseous species, the appearance of reflections belonging to triclinic Ti(IV)p is detected. Above 658 K, only the Ti(IV)p phase is observed.

**Figure 10:**
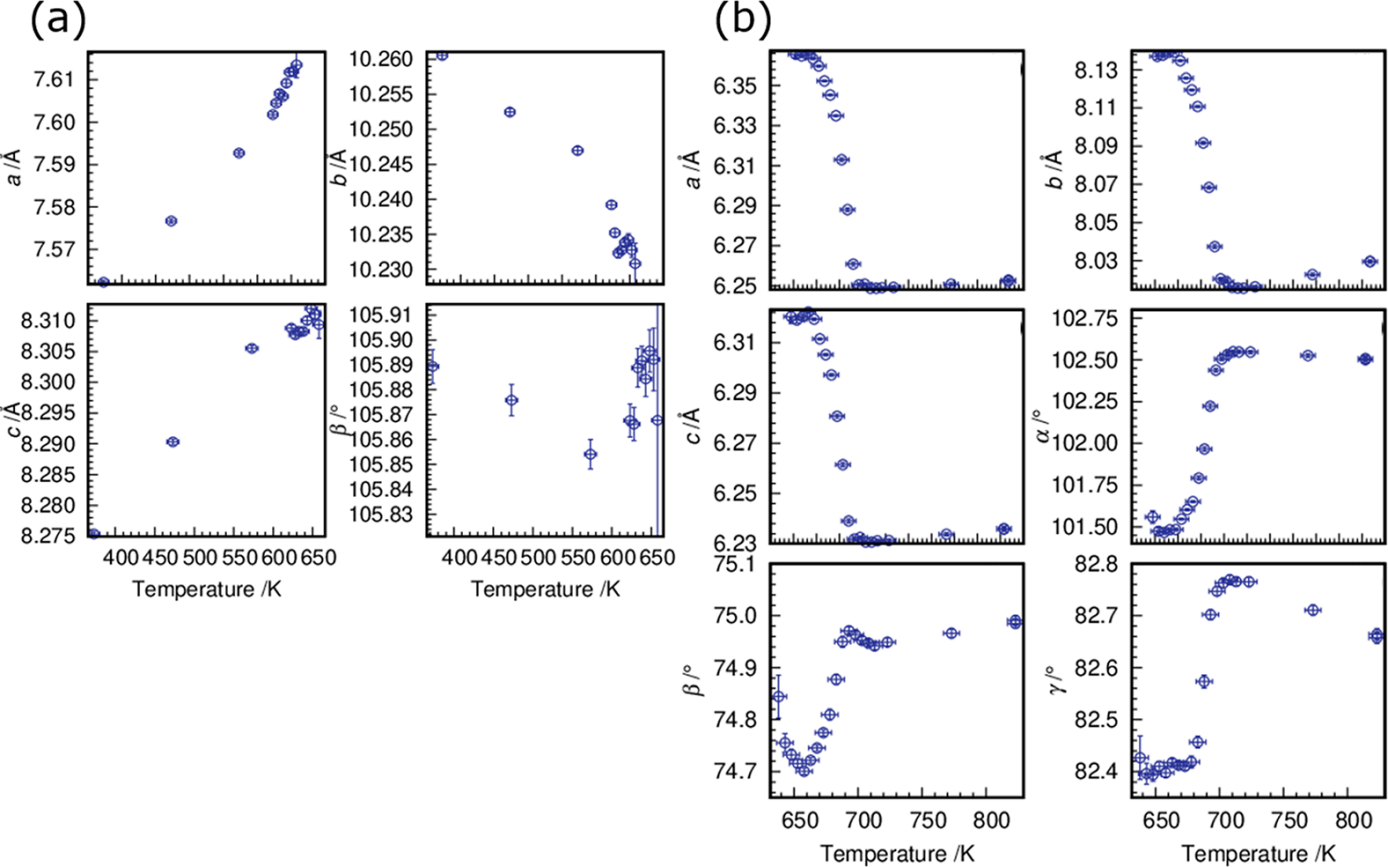
(a) Evolution of the unit cell parameters of Ti(III)p with temperature: the lattice parameters *a*, *b*, and *c* and the angle β. (b) Plus the unit cell parameters of Ti(IV)p: the lattice parameters *a*, *b*, and *c* and the angles α, β, and γ.

The evolution of the lattice parameters of Ti(III)p with temperature reveals the formation of the high-temperature phase Ti(IV)p (Figure 10a,b). In particular, the lattice parameter *b* and the angle β are sensitive indicators for the phase transformation. The lattice parameters of Ti(IV)p scatter up to 633 K (Figure 10b). Above this temperature, all metric parameters show a sudden increase to a more or less stable value. The sole exception is the angle γ, which decreases after remaining at a small plateau (698 < *T* < 723 K). The TD evolution of the metric parameters of Ti(IV)p implies a subsequent relaxation and order of the newly formed phase. As mentioned above, the TGA–MS data of the reaction of Ti(III)p to Ti(IV)p exhibit two distinct endothermic signals.^26^ These signals can be correlated to two events visible in the *in situ* PXRD data: first, thermal decomposition of NH_4_^+^ to NH_3_ and H_2_ and, second, subsequent relaxation of the Ti(IV)p structure. Stegmann et al. observed in the DSC data two exothermic signals (*T* = 723 and 763 K) that can be correlated to the two-step reaction.^26^ The differences in temperatures between the X-ray diffraction and DSC experiments are caused by different instrumental setups.

Also *in situ* Raman spectra were recorded upon heating Ti(III)p to 823 K in an inert atmosphere (Figure S3). From ambient temperature to 628 K, the characteristic Raman modes of Ti(III)p are observed. In the temperature range from 633 to 643 K, the intensities of all modes decrease. The *in situ* PXRD data reveal that in this temperature window thermal decomposition of NH_4_^+^ together with the formation to Ti(IV)p proceeds. At higher temperatures (*T* > 643 K), then the characteristic modes of Ti(IV)p are observed.

**Figure 11:**
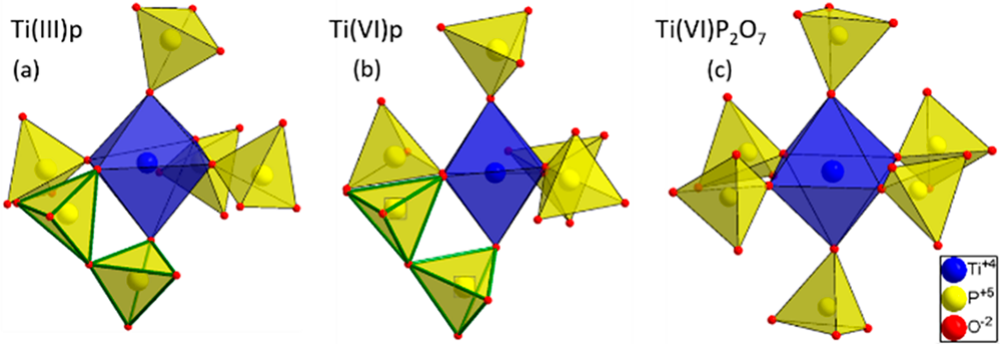
Second coordination spheres of titanium cations within (a) Ti(III)p, (b) Ti(IV)p, and (c) TiP_2_O_7_. The [P_2_O_7_]^4–^ group, which is connected via two coordination sites, is marked in green.

In a subsequent *in situ* PXRD experiment performed at 823 K in synthetic dried air (Figure S4), Ti(IV)p was retained for 3 h. Under these conditions, no reaction of Ti(IV)p to cubic TiP_2_O_7_ could be detected. However, during the synthesis in air, a phase transformation from Ti(IV)p to TiP_2_O_7_ was observed. The major structural difference of Ti(III)p and Ti(IV)p compared to TiP_2_O_7_ is in the connection of the [P_2_O_7_]^4–^ groups. In the Ti(III)p structure, each Ti^III^ is connected to five [P_2_O_7_]^4–^ groups, all as monodentate ligands except one, which is connected via two bridging oxygen atoms (Figure 11). This local configuration is stable during the phase transformation to Ti(IV)p upon annealing in inert atmospheres. The phase transformation of either Ti(III)p or Ti(IV)p to TiP_2_O_7_ would necessarily cause a change in the connectivity because, in the latter structure, the titanium(IV) cation is surrounded by six monodentate [P_2_O_7_]^4–^ groups. Because the phase transformation to TiP_2_O_7_ proceeds in an ambient atmosphere but not in dried synthetic air, we assume that the presence of humidity is required for this change in coordination.

## Conclusion

The structure determination of two novel titanium pyrophosphate structures, Ti(III)p and Ti(IV)p, has been successfully carried out by combining complementary diffraction and spectroscopic techniques. By this stepwise approach, first, the average local atomic structure and then the bulk crystal structure were determined. Previous studies of Ti(III)p discussed the chemical composition NH_4_TiP_2_O_7_.^26^ The XPS data indicated tri- and tetravalent titanium cations on the surface of the compound, while for Ti(IV)p, only tetravalent titanium was observed. By total scattering experiments with subsequent PDF analysis, the presence of only trivalent titanium species was confirmed in the bulk structure of Ti(III)p. The tetravalent species observed by XPS originate from surface oxidation of the Ti(III)p sample. The PDF data of Ti(IV)p show tetravalent species in accordance with the XPS data. The coordination polyhedra were studied via Raman spectroscopy and total scattering experiments, resulting in primary building units similar to those known for cubic TiP_2_O_7_. All structures consist of pyrophosphate units and TiO_6_ octahedra. In the case of Ti(III)p, additional NH_4_^+^ cations and highly distorted TiO_6_ octahedra are observed.

The average structure models for both phases (Ti(III)p and Ti(IV)p) were derived by an ab initio structure solution via *simulated annealing*. For Ti(III)p, a monoclinic structure in *P*2_1_/*c* with highly distorted TiO_6_ octahedra connected to five pyrophosphates, four as monodentate ligands, and one bidentate ligand, was determined. This secondary coordination sphere causes the formation of [TiP_4_O_12_] layers and one-dimensional channels along the crystallographic *c* direction. The channels are occupied by NH_4_^+^ cations. The subsequent Rietveld refinement results in reasonable bond distances as well as angles. The observed residual electron density as well as a mismatch in the PDF refinement of atom pair correlations above 4 Å indicates stacking disorder and partial blocking of the channel system. Nevertheless, the model is describing the average Ti(III)p structure quite well, as additionally proven by good agreement of the experimental Raman shifts with the theoretical ones. The structure of Ti(IV)p crystallizes in the space group *P*1̅. The structure solution results in the same Ti–O–P network with [TiP_4_O_12_] layers and one-dimensional channels. Unlike the Ti(III)p structure, the channels are empty in the Ti(IV)p structure. The release of NH_3_ from the structure as well as the oxidation of tri- to tetravalent titanium causes structure relaxation, detectable in the higher symmetry of the TiO_6_ octahedra and a more regular channel dimension. Subsequent Rietveld refinement and the fitting of the PDF data show good agreement of the respective simulated data from the model and the measured data. Besides, the obtained model shows reasonable bond distances and angles. The obtained structure model for Ti(IV)p was validated via Raman and NMR spectroscopy, as well as by DFT calculations.

The *in situ* Raman and PXRD data reveal that, in inert N_2_ atmosphere, Ti(III)p reacts to Ti(IV)p at 633 K. Above 633 K, a contraction of the structure correlated with the decomposition of NH_4_^+^ to NH_3_ and the simultaneous redox reaction 2H^+^ + 2Ti^3+^ → H_2_ + 2Ti^4+^ is observed. Besides, the formation of Ti(IV)p is detected at 633 K. The evolution of the metric parameters of Ti(IV)p with temperature implies relaxation and ordering of the newly formed phase at 723 K. The reaction of Ti(IV)p to cubic TiP_2_O_7_ at 823 K, which readily proceeds in ambient air, could not be detected in dried synthetic air, showing the temperature stability of the local configuration in the absence of water.

The proton conductivity of the newly synthesized phosphates Ti(III)p and Ti(IV)p seems to be based on the Grotthus mechanism. The specific proton conductivity and the activation energy of the proton migration of Ti(III)p belong to the highest and lowest, respectively, ever reported for this class of materials and indicate its potential application as a proton-conducting electrolyte for electrochemical devices like fuel cells and water electrolyzers, working in the intermediate temperature range.

## Supporting Information Available

Geometrical information on the rigid bodies used for refinement, fractional coordinates of the solved structure models, a detailed discussion of the resulting geometrical parameters (bond angles and bond distances), residual electron densities, detailed discussion of the modeling of the PDF data, additional TD Raman data of Ti(III)p under an inert atmosphere, and the TD PXRD data of Ti(IV)p collected at 823 K in synthetic air plus the TD proton conductivities of Ti(III)p and Ti(IV)p (PDF)

## Accession Codes

CCDC 2105122 and 2105123 contain the supplementary crystallographic data for this paper. These data can be obtained free of charge via www.ccdc.cam.ac.uk/data_request/cif, or by emailing data_request@ccdc.cam.ac.uk, or by contacting The Cambridge Crystallographic Data Centre, 12 Union Road, Cambridge CB2 1EZ, UK; fax: +44 1223 336033.
