# Supplementary material for: Crystal Structures of Two Titanium Phosphate-Based Proton Conductors: Ab Initio Structure Solution and Materials Properties
Source: Inorg Chem. 2021 Nov 22;61(5):2379–90. doi: 10.1021/acs.inorgchem.1c02613 (PMC8826274; doi:10.1021/acs.inorgchem.1c02613)
Supplement: Supplementary file 1 — ic1c02613_si_002.pdf [file ic1c02613_si_002.pdf]

Supporting Information

Crystal structures of two titanium phosphate-based proton conductors: ab initio structure solution and materials properties

Hilke Petersen[1], Niklas Stegmann[1], Michael Fischer[2,3], Bodo Zibrowius[1], Ivan Radev[4], Wladimir Philippi[4], Wolfgang Schmidt[1], Claudia Weidenthaler[1]*

1 Max-Planck-Institut für Kohlenforschung, Heterogeneous Catalysis, Kaiser-Wilhelm-Platz 1, 45470 Mülheim an der Ruhr, Germany

2 MAPEX Center for Materials and Processes, University of Bremen, 28334 Bremen, Germany

3 Crystallography/Geosciences, University of Bremen, Klagenfurter Straße, 28359 Bremen, Germany

4 The Hydrogen and Fuel Cell Center - ZBT GmbH, Carl-Benz-Straße 201, 47057 Duisburg, Germany

Corresponding Author

Claudia Weidenthaler*. Max-Planck-Institut für Kohlenforschung, Heterogeneous Catalysis, Kaiser-Wilhelm-Platz 1, 45470 Mülheim an der Ruhr, Germany;ORCID 0000-0003-3006-1333; Email: weidenthaler@mpi-muelheim.mpg.de

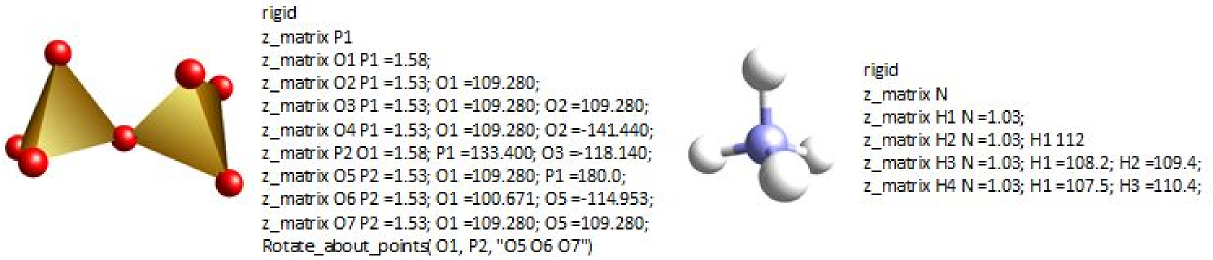

Figure S1. Rigid body units of the $P_2O_7^{4-}$ and NH_4^+ groups.

Table S1. Fractional atomic coordinates and isotropic displacement parameter of Ti(III)p.

$NH_4TiP_2O_7$						
Monoclinic, *P*2$_1$/c (14)						
M:	959.446 $gmol^{-1}$					
a:<br>*b:*<br>*c:*<br>*β:*	7.5465(2) Å<br>10.2547(2) Å<br>8.2591(2) Å<br>105.891(3)°					
V:	614.72(1) $Å^3$					
Z:	4					
site	Wyckoffposition	x	y	z	occupancy	B_{iso} /Å
Ti1	4e (*x, y, z*)	0.2531(9)	0.3909(5)	0.2378(8)	1	2.5(1)
P1	4e (*x, y, z*)	0.361(1)	0.0843(7)	0.183(1)	1	0.49(9)
P2	4e (*x, y, z*)	0.062(1)	0.1232(8)	0.313(1)	1	0.49(9)
O1	4e (*x, y, z*)	0.184(2)	0.054(1)	0.222(2)	1	0.49(9)
O2	4e (*x, y, z*)	0.501(2)	-0.019(1)	0.235(2)	1	0.49(9)
O3	4e (*x, y, z*)	0.336(2)	0.088(1)	-0.009(2)	1	0.49(9)
O4	4e (*x, y, z*)	0.412(2)	0.237(1)	0.21(2)	1	0.49(9)
O5	4e (*x, y, z*)	0.045(2)	0.269(1)	0.271(1)	1	0.49(9)
O6	4e (*x, y, z*)	0.164(2)	0.112(1)	0.497(1)	1	0.49(9)
O7	4e (*x, y, z*)	-0.149(1)	0.089(1)	0.245(1)	1	0.49(9)

N	4e (*x, y, z*)	0.66(2)	0.315(1)	0.047(2)	1	3(5)
H1	4e (*x, y, z*)	0.76(3)	0.27(2)	0.14(1)	1	3(5)
H2	4e (*x, y, z*)	0.66(1)	0.29(1)	-0.073(6)	1	3(5)
H3	4e (*x, y, z*)	0.53(2)	0.28(3)	0.05(2)	1	3(5)
H4	4e (*x, y, z*)	0.66(4)	0.411(4)	0.08(2)	1	3(5)

Table S2. Fractional atomic coordinates and isotropic or equivalent isotropic displacement parameter of Ti(IV)p.

TiP_2O_7						
Triclinic, $P\overline{1}$ (2)						
M:	443.646 gmol^{-1}					
a:<br>*b:*<br>*c:*<br>*α:*<br>*β:*<br>γ:	6.2292(1) Å<br>7.9483(1) Å<br>6.2065(1) Å<br>102.804(2)°<br>74.8277(18)°<br>83.203(2)°					
V:	284.08(1) Å^3					
Z:	2					
Site	Wyckoffposition	x	y	z	occupancy	Biso /Å^2

Ti1	2i (*x, y, z*)	0.311(7)	0.3110(5)	0.376(8)	1	0.52(4)
P1	2i (*x, y, z*)	0.243(1)	0.2431(8)	0.509(1)	1	0.52(4)
P2	2i (*x, y, z*)	0.531(1)	0.5308(8)	0.187(1)	1	0.52(4)
O1	2i (*x, y, z*)	0.657(2)	0.6573(1)	-0.067(2)	1	0.52(4)
O2	2i (*x, y, z*)	0.37(1)	0.3702(1)	0.247(1)	1	0.52(4)
O3	2i (*x, y, z*)	0.239(1)	0.2394(1)	0.498(2)	1	0.52(4)
O4	2i (*x, y, z*)	0.365(2)	0.3650(1)	0.663(2)	1	0.52(4)
O5	2i (*x, y, z*)	-0.008(2)	0.0078(1)	0.579(1)	1	0.52(4)
O6	2i (*x, y, z*)	0.715(1)	0.7154(1)	0.319(2)	1	0.52(4)
O7	2i (*x, y, z*)	0.384(1)	0.3836(1)	0.235(1)	1	0.52(4)

Structure evaluation. The obtained Ti-O bond distances (Table S3) are typical for Ti(+III)-O distances ($Ti(PO_3)_3$: Ti-O=203.3(2)-202.9(2) pm), fitting well to the results obtained from XPS and PDF analysis.[18] The TiO_6 octahedron in the Ti(III)p structure shows a high distortion with bond distances between 1.98(1) – 2.08(1) Å and O_P-Ti-O_A angles (O_P = planar oxygen atoms; O_A = axial oxygen atoms) ranging 81.6(1)-103.8(1)° and an O_A-Ti-O_A angle of 163.5(1)°, reflecting the Raman results. The P-O bond distances in the $[P_2O_7]^{4-}$ groups imply regular tetrahedra with an average P-O bond length of 1.51(4) Å. Norberg et al. observed similar P-O

bond distances (r(P-O) = 1.489(1) – 1.506(7) Å) in the cubic TiP_2O_7 structure.[24] The P-O-P angle of 135.07(1)° are smaller compared to values obtained from Rietveld refinement of TiP_2O_7 (() = 139(1)-145(1)).[23] Sanz et al. introduced the formula $180°\text{-}2\cos^{-1}(d_{P\text{-}O1}/158.8)$ to estimate the true P-O-P angle.[23] According to this expression, P-O-P is equal to 139(5)° fitting well to the obtained values for TiP_2O_7. Besides, the intramolecular P-P distance 2.74(1) Å of the $P_2O_7^{4-}$ is shortened in comparison to the PDF results (2.881(2) Å (Figure 3a,b).

The intramolecular P-P distances are strongly correlated to P-O-P angles, therefore the deviation between the results of the Rietveld refinement and PDF may be due to the smaller P-O-P angles obtained from the XRPD data.

Table S3. Selected geometric parameters of Ti(III)p.

Bond	Bond length [Å]	Bond	Bond length [Å]	Angle	Angle /°
P1-O1[a]	1.48(2)	P1-P2	2.74(1)	P1-O1-P2	135.07(1)
P1-O2	1.44(2)				
P1-O3	1.55(2)	Ti-O2	2.01(1)	O5TiO4	87.4(1)
P1-O4	1.57(2)	Ti-O3	2.00(2)	O5TiO6	90.8(1)
P2-O1[a]	1.49(2)	Ti-O4	2.09(1)	O5TiO7	103.8(1)
P2-O5	1.48(2)	Ti-O5	2.10(1)	O5TiO3	87.52(1)
P2-O6	1.50(1)	Ti-O6	1.98(1)	O5TiO2[b]	163.5(1)
P2-O7	1.54(1)	Ti-O7	2.13(1)	O2TiO4	81.6(1)
				O2TiO6	99.82(1)
				O2TiO7	88.4(1)
				O2TiO3	82.7(1)

[a] O1 is the bridging oxygen atom in the $[P_2O_7]^{4-}$ group

[b] O2 and O5 are the axial oxygen atoms in $[TiO_6]^{9-}$

Structure evaluation. The terminal P-O bond distances ($P_{1,2}$-O_x; x=2-7: $r_{P_{1,2}O_x}$=1.54(2) Å) of the $P_2O_7^{4-}$ groups are comparable to Ti(III)p. The bridging P-O bonds (r(P1,2-O1) = 1.59(1) Å) on the other hand are significantly elongated (Table S3 and Table S4). In the superstructure of cubic TiP_2O_7 also an elongation of the bridging P-O bonds was detected.[23, 24] Especially the $P_2O_7^{4-}$ groups on general crystallographic positions show a comparable average bond length of

1.584(8) Å and 1.575(5) Å.[23, 24] In addition, the P-P distances in the $P_2O_7^{4-}$ groups ($r_{P_1P_2}$ =2.83(1) Å) are elongated in comparison to Ti(III)p ($R(P_1P_2)$ = 2.74(1) Å). The Ti-O bond lengths are shorter compared to Ti(III)p mirroring the oxidation of Ti^{3+} to Ti^{4+}. The average bond distance of 1.90(4) Å, fits well to the average Ti-O bond length in cubic TiP_2O_7 1.92(1) Å and 1.92 Å.[23, 24] Besides, Raman data of Ti(IV)p indicate a more regular TiO_6 octahedron comparable to TiP_2O_7. Especially, the O_P-Ti-O_A angles ranging from 89.4(5) to 90.4(5)° and the O_A-Ti-O_A angle of 177.5(5)° indicates a higher symmetry.

Table S4. Selected geometric parameters of Ti(IV)p.

Bond	Bond length [Å]	Bond	Bond length [Å]	Angle	Angle /°
P1-O1[a]	1.59(1)	P1-P2	2.83(1)	P1-O1-P2	152.9(1)
P1-O2	1.52(1)				
P1-O3	1.57(1)	Ti-O2	1.93(1)	O6TiO7	90.0(5)
P1-O4	1.55(1)	Ti-O3	1.84(1)	O6TiO4	87.1(5)
P2-O1[a]	1.59(1)	Ti-O4	1.94(1)	O6TiO3	90.3(5)
P2-O5	1.57(1)	Ti-O5	1.85(1)	O6TiO2	89.9(5)
P2-O6	1.51(1)	Ti-O6	1.96(1)	O5TiO6[b]	177.5(5)
P2-O7	1.52(1)	Ti-O7	1.90(1)	O5TiO7	89.4(5)
				O5TiO4	90.4(5)

				O5TiO3	90.5(5)
				O5TiO2	92.5(5)

[a] O1 is the bridging oxygen atom in the $[P_2O_7]^{4-}$ group

[b] O5 and O6 are the axial oxygen atoms in $[TiO_6]^{9-}$

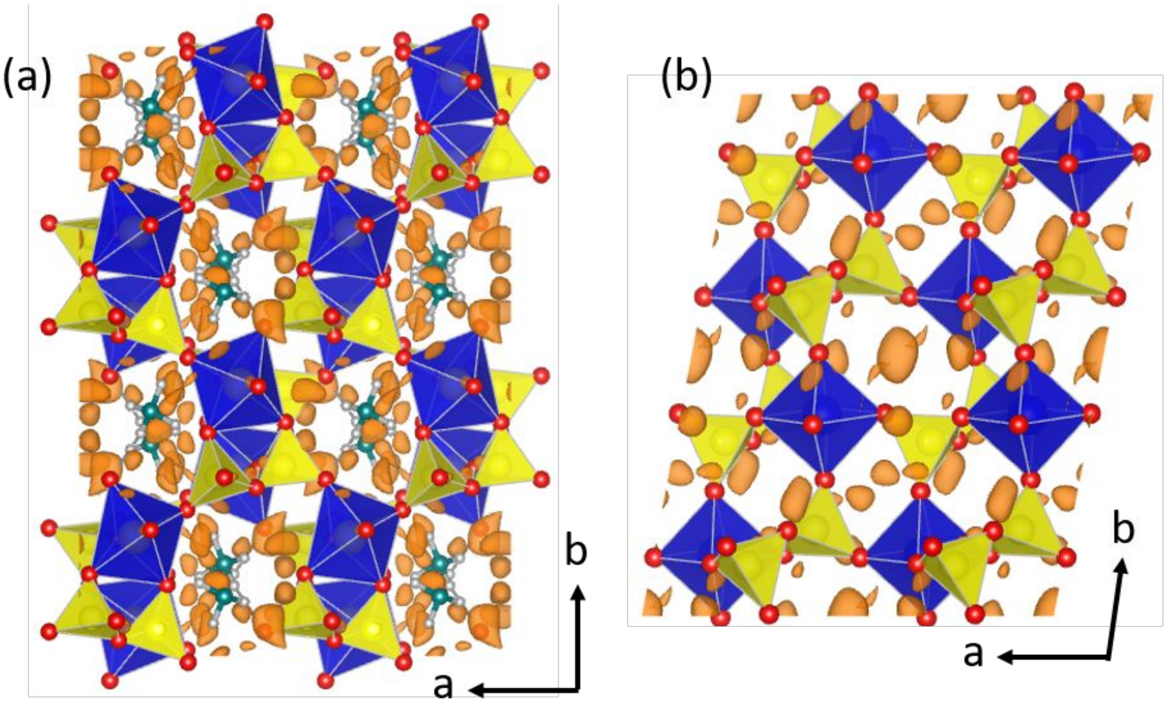

Figure S2. Residual electron density in the channel system of (a) Ti(III)p and (b) Ti(IV)p.

Modeling of PDF data. Modeling of the PDF data gives an average P-O bond distance of 153(2) pm; the average refined Ti-O bond distance is 2.03(8) Å. The calculated O-O pair correlations match the measured data but their probability is underestimated. For both the P-P distances in the pyrophosphate groups as well as the Ti-P distances, the measured and refined correlations are in good agreement. For longer distances above r ~ 4 Å the deviations between

model and measurement increase. The mismatch for larger distances indicates stacking faults in the [TiP_4O_{12}]-layers resulting in a partial blocking of the one-dimensional channel structure. Especially the bond distances between ~400 to 600 Å can be correlated to the dimensions of the channel structure of Ti(III)p (d_{min} = 3.67(1) Å and d_{max} = 5.14(1) Å). In this range of bond distances, the refinement shows a significant disagreement between measured and calculated data, observable in the increased scattering of the difference curve (Figure 3a) implying the partial collapse of the channel structure. A partial collapse of the channel structure could be caused by stacking faults of the [TiP_4O_{12}]-layers (Figure 5b) or local differences in NH_4^+ positions as well as of the occupancy.

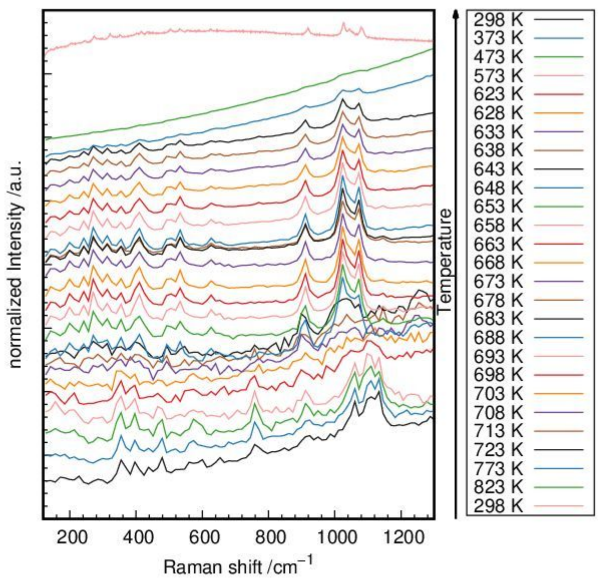

Figure S3. Temperature-dependent Raman spectra of Ti(III)p measured under inert atmosphere (N_2).

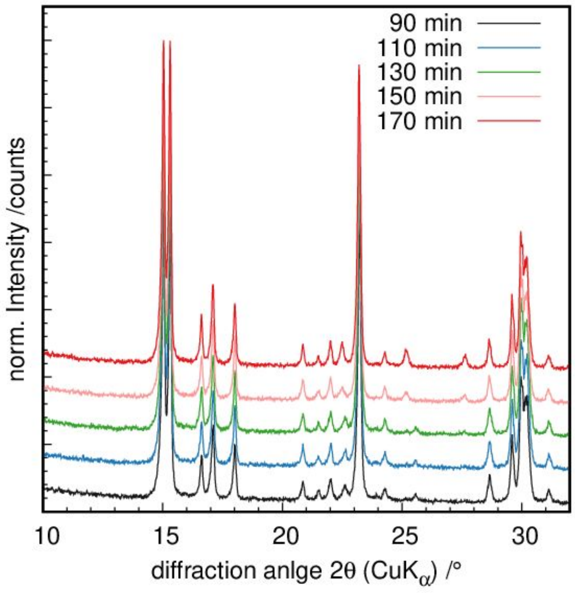

Figure S4. Time-dependent in situ XRPD data of Ti(IV)p collected at 823 K in synthetic air.

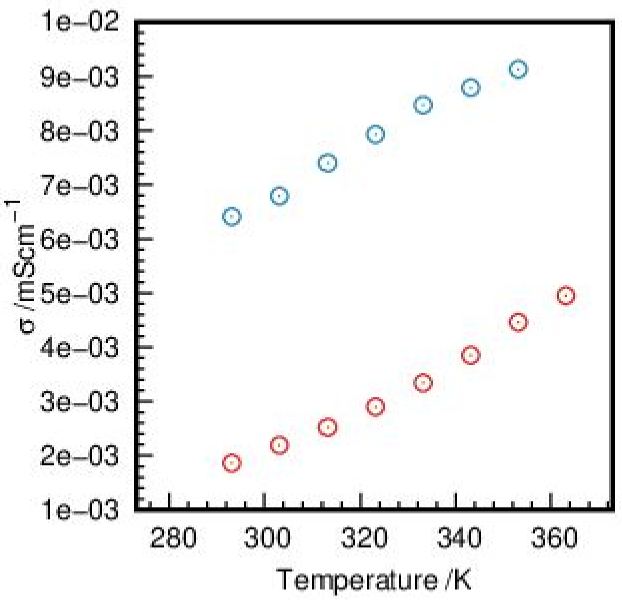

Figure S5. Proton conductivities of Ti(III)p (blue) and Ti(IV)p (red) as a function of temperature under fully hydrated conditions.
